# Supplementary material for: Integrated 3D imaging of FFPE lung tissue combining microCT, light and electron microscopy allows for contextualized ultrastructural and histological analysis
Source: Sci Rep. 2025 May 28;15:18656. doi: 10.1038/s41598-025-02770-w (PMC12120005; doi:10.1038/s41598-025-02770-w)
Supplement: Supplementary file 1 — Supplementary Information. [file 41598_2025_2770_MOESM1_ESM.docx]

## Supplemental Material

# Integrated 3D Imaging of FFPE Lung Tissue Combining MicroCT Light and Electron Microscopy Allows for Contextualized Ultrastructural and Histological Analysis

Johanna Reiser, Jonas Albers, Angelika Svetlove, Mara Mertiny, Felix K.F. Kommoss, Constantin Schwab, Anna Schneemann, Giuliana Tromba, Irene Wacker, Ronald E. Curticean, Rasmus R. Schroeder, Hans-Ulrich Kauczor, Mark O. Wielpütz, Christian Dullin, and Willi L. Wagner

|  | PBI based Synchrotron microCT | | microCT | |
| --- | --- | --- | --- | --- |
| Setup | SYRMEP white beam setup | | Zeiss 620 Versa | |
| Beam Energy / tube voltage | average energy 20 keV | | 40 kV (8W) | |
| Filtering | 1mm Si | | None | |
| Flux | ~10^11^ ph/mm^2^ s | | § | |
| Detector | Hamamatsu Orca Flash sCMOS, 2048×2048 px^2^ - pixel size of 6.5×6.5 μm^2^ | | proprietary detector | |
| Exposure time | 50 ms | 100 ms | | 5000 ms |
| Scan geometry | centered 180° | | centered 360° | |
| Projections | 1800 | 1800 | | 5001 |
| Total scanning time | 90 s | 180 s | | 9.3 h |
| Objective lens / magnification | flexible zoom | flexible zoom | | 4x |
| Reconstructed voxel size | 4.5x4.5x4.5 µm^3^ | 2x2x2 µm^3^ | | 1x1x1 µm^3^ |
| Scanned volume [px] | 2048x2048x778 | 20248x2048x1750 | | 1031x1031x1031 |
| Sample condition | unstained 4mm punch biopsy of a standard FFPE lung tissue block | | | |
| Sample-to-Detector distance | 45 cm | 15 cm | | 4 cm |
| Multiple scans | 3 vertically stacked (increment 3mm) | | No | |

§ where are no x-ray flux values available. The flux is highly dependent on the focal spot size and the tube current. Typically, the flux in a microCT is about 3 orders of magnitude smaller than for synchrotron-CT
